# Preparation, Structural Characterization, and Hypoglycemic Activity of Dietary Fiber from Sea Buckthorn Pomace

**DOI:** 10.3390/foods13223665

**Published:** 2024-11-18

**Authors:** Qi Xiao, Liting Yang, Jingjing Guo, Xiyu Zhang, Yu Huang, Qun Fu

**Affiliations:** 1 College of Life Sciences, Northeast Forestry University, Harbin 150040, China; 18560682059@163.com (Q.X.); yangliting0131@163.com (L.Y.); 13349323590@163.com (J.G.); z_xy0214@outlook.com (X.Z.); huangyxxxi@163.com (Y.H.); 2 Key Laboratory of Forest Food Resource Utilization of Heilongjiang Province, Harbin 150040, China

**Keywords:** *Hippophae rhamnoides* L., dietary fiber, ultrasound-bound enzyme assay, hypoglycemic enzyme, structure

## Abstract

Sea buckthorn pomace is often discarded as a by-product during the sea buckthorn processing stage. Consequently, its richness in dietary fiber is usually overlooked. In this study, soluble dietary fiber (SDF) and insoluble dietary fiber (IDF) were extracted from sea buckthorn pomace using ultrasound combined with the enzyme method. The optimal values of the independent variable were determined by a combinatorial design and a response surface optimization test with SDF/IDF as the dependent variable, prepared as follows: 5% enzyme addition, ultrasonic power of 380 W, enzymatic time of 30 min, and alcoholic precipitation liquid ratio of 4:1. Under these conditions, the SDF/IDF ratio was 17.07%. The structural characterization and hypoglycemic activity of the two dietary fibers were then compared. The results show that two dietary fibers have respective structures and functional groups of fibers. SDF was less crystalline than IDF, and its structure was looser. Furthermore, the hypoglycemic activity of SDF was significantly better than IDF’s (*p* < 0.05). The glucose adsorption capacity of SDF was 1.08–1.12 times higher than that of IDF. SDF inhibited *α*-amylase and *α*-glucosidase by 1.76 and 4.71 times more than IDF, respectively. These findings provide a reference for improving the utilization of sea buckthorn processing by-products.

## 1. Introduction 

Diabetes mellitus is a metabolic disease characterized by hyperglycemia. In recent years, hyperglycemia is increasingly occurring in the population as a result of high-sugar and high-fat dietary patterns [1]. According to the International Diabetes Federation, there were 537 million diabetic patients globally in 2021. Dietary fiber (DF) has physiological benefits such as inhibiting weight gain and lowering blood glucose [2], and so it is referred to as the “seventh major nutrient” [3]. DF can be classified as soluble dietary fiber (SDF) and insoluble dietary fiber (IDF) based on its solubility in water. IDF includes hemicellulose, cellulose, lignin, etc. SDF generally includes pectin, *β*-glucans, galactomannans, and fructans [4].

IDF and SDF have significantly different physiological functions. For example, IDF mainly acts on the intestine to produce a mechanical peristaltic effect [5], while SDF mainly participates in metabolic processes, such as glucose and lipid metabolism. In addition, the ratio of SDF and IDF is closely related to the effectiveness of the physiological function of DF, which makes it an important index for evaluating the quality of DF. Generally, the higher the SDF/IDF ratio, the better the quality of the DF and the better its physiological efficacy [6].

Sea buckthorn (*Hippophae rhamnoides* L.) is globally recognized as an economic-protective plant. It can bring a series of environmental benefits such as soil and water conservation, wind-breaking and sand-fixing. [7]. It is distributed in 53 countries around the world, with China having the wealthiest sea buckthorn resources [8]. In China, the annual output of its fruits reaches 250,000 metric tons, and the total annual industrial output value is USD 337–365 million [9]. Sea buckthorn pomace is the main by-product from the processing of sea buckthorn. The pomace rate of sea buckthorn can be more than 50%. The proportion of SDF in the total dietary fiber (TDF) of sea buckthorn pomace (13.06%) [10] is significantly more than SDF in other plant pomace, such as grape pomace (6.10%) [11] and pineapple pomace (9.50%) [12]. Therefore, sea buckthorn can be potentially applied in the development of high-quality fiber foods. However, sea buckthorn pomace is usually discarded as processing waste, leading to environmental pollution and the waste of biological resources [13].

Despite its economic potential and environmental benefits, the current research on sea buckthorn pomace is relatively limited. Regarding the extraction method, researchers primarily use physical, chemical and biological techniques for the extraction of DF. Each method, however, faces its own technical challenges, such as chemical residues, low efficiency, and time consumption problems [14,15]. Thus, some researchers have used combinational methods to address the above shortcomings. The combination of different methods can improve the functional properties of DFs, aside from the extraction efficiency [16]. For instance, the DF from kinnow peels obtained by combined ultrasound and enzymatic treatment was higher than that obtained by only ultrasound treatment [17]. In Tang’s study, the structure and function of DF in bamboo shoots obtained by combined ultrasound and enzymatic treatment were superior to those of the enzyme treatment only [18].

Currently, there are few studies on extracting high-quality DF from sea buckthorn pomace by an ultrasound–enzyme method. SDF/IDF was targeted in this study, and the optimal extraction process of the ultrasound–enzyme method was determined by combinatorial design and optimization tests. The objective of this study is to develop high-quality, high-yield, and easy-to-produce dietary fiber. The structural characteristics and hypoglycemic effects of sea buckthorn pomace SDF and IDF under this method were also comparatively analyzed. This research will provide the theoretical groundwork for the development of hypoglycemic products and the sustainable use of agro-processing by-products.

## 2. Materials and Methods

### 2.1. Materials

Sea buckthorn pomace, provided by Heilongjiang Academy of Agricultural Sciences (Harbin, China), was the by-product of sea buckthorn fresh fruit juice extraction.

Chemicals used were as follows: 4-nitrophenyl-*β*-D glucopyranoside (PNPG, ≥98%), acarbose (≥95%), 3,5-dinitrosalicylic acid (DNS), *α*-glucosidase (700,000 U/mL) and *α*-amylase (5000 U/g) were purchased from Shanghai Yuanye Bio-Technology Co. (Shanghai, China); cellulase (>100 U/mg) was purchased from Shanghai Lanji technology development Co. (Shanghai, China); petroleum ether, trichloromethane, 1-Butanol and sodium hydroxide were purchased from Tianjin Fuyu Fine Chemical Co. (Tianjin, China); ethanol absolute, sodium carbonate, glucose and soluble starch were purchased from Tianjin Tianli Chemical Reagent Co. (Tianjin, China).

### 2.2. Extraction and Optimisation of DF

**Figure 1:**
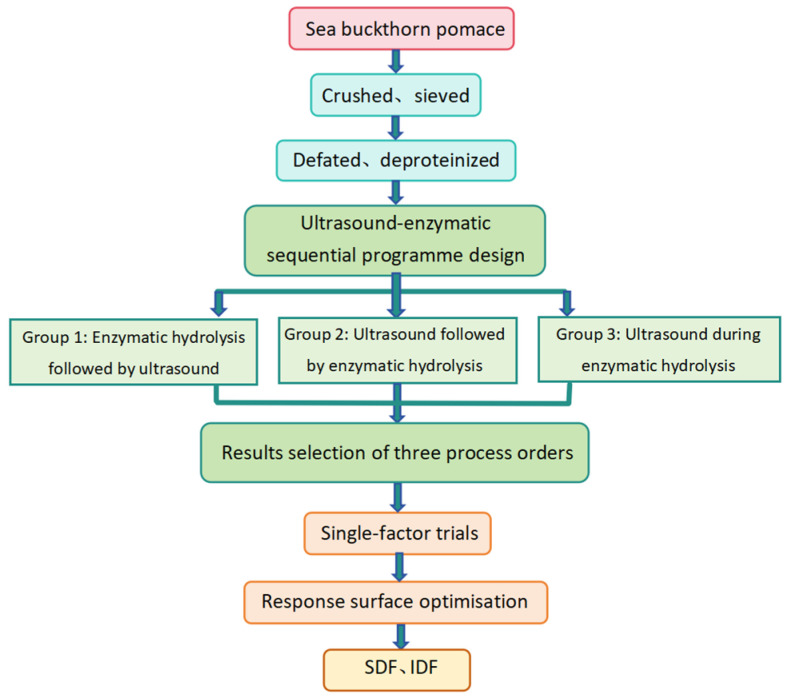
Process for DF extraction from sea buckthorn pomace.

Both ultrasound and enzyme hydrolysis are effective ways of extracting DF (Figure 1). The order of application of the two may have an impact on the conversion and dissolution between SDF and IDF in pomace. Therefore, the application order of ultrasound and enzymatic hydrolysis was firstly investigated. After determining the optimal combination order, the subsequent optimization tests were carried out.

#### 2.2.1. Ultrasound–Enzymatic Sequential Program Design Tests

Sea buckthorn pomace was dried at 50 °C, crushed, and sieved through 80 mesh. The pomace powder was defatted in petroleum ether. The filter residue was washed with 60% ethanol and dried. Then, the defatted pomace was added to chloroform: n-butanol at 4:1 (*v*/*v*), vigorously shaken for 30 min to deproteinize, and dried. The group experiments were carried out by taking 5 g of the treated pomace powder and adding 100 mL of distilled water as follows: (Group 1) Enzymatic hydrolysis followed by ultrasound. The samples were first adjusted pH to 4.8, added to 5% (*w*/*w*) cellulase, and subjected to enzymatic hydrolysis at 50 °C for 30 min. Finally, the samples were placed under sonication (NP-PS-80AL, North Point Scientific Co., Ningbo, Zhejiang Province, China) at 50 °C and 380 W for 40 min [19]. (Group 2) Ultrasound followed by enzymatic hydrolysis. The samples were first subjected to sonication at 50 °C and 380 W for 40 min, then the pH was adjusted to 4.8, and 5% cellulase was added. Finally, the samples were subjected to enzymatic hydrolysis at 50 °C for 30 min. (Group 3) Ultrasound during enzymatic hydrolysis. The pH of the samples was adjusted to 4.8, and 5% cellulase was added, and finally the samples were subjected to 50 °C and 380 W for 70 min [20].

After the reaction of each group was completed, the enzyme was inactivated at 95 °C for 20 min and centrifuged at 19,200× *g* (5000 rpm) at 25 °C for 15 min (TG16G, Hangzhou Chuanheng Experimental Instrument CO., Hangzhou, China). After being washed with distilled water at 60 °C, the precipitate was centrifuged once more. The filtrate was freeze-dried to obtain IDF, calculated as follows:

(1) $$The\;yield\;of\;IDF\left(\%\right)=\frac{W_{1}}{W_{0}}\times 100$$

where *W*_1_ is the weight of IDF (g), and *W*_0_ is the weight of sea buckthorn pomace powder (g).

The supernatant was combined with the washing solution. The mixture was concentrated to 1/3 of the original volume. The concentrate was precipitated by alcohol for 6 h before being centrifuged. SDF was obtained by freeze-drying the precipitate, calculated as follows:

(2) $$The\;yield\;of\;SDF\left(\%\right)=\frac{W_{2}}{W_{0}}\times 100$$

where *W*_2_ is the weight of SDF (g), and *W*_0_ is the weight of sea buckthorn pomace powder (g).

The amount of SDF/IDF (%, *w*/*w*) obtained from each set of experiments was compared, and the combination with the highest SDF/IDF was selected to carry out single-factor trials.

#### 2.2.2. Optimization of DF Extraction from Sea Buckthorn Pomace

Single-factor trials were conducted to investigate the effects of cellulase addition (3, 4, 5, 6, and 7% (*w*/*w*)), enzymatic hydrolysis time (20, 30, 40, 50, and 60 min), alcoholic precipitation liquid ratio (2:1, 3:1, 4:1, 5:1, and 6:1 (*v*/*v*)), and ultrasonic power (230, 280, 330, 380, and 430 W) in the SDF/IDF extracted from pomace. Based on the results of the single-factor test, the four factors mentioned above were used as independent variables and SDF/IDF as dependent variables. A four-factor, three-level experimental design was carried out according to the Box–Behnken experimental principle, and the optimal parameters of the extraction process were determined by the response surface optimization method. The relationship between the independent and dependent variables followed Equation (3).

(3) $$Y=\beta_{0}\;+\beta_{1}A+\beta_{2}B+\beta_{3}C+\beta_{4}D+\beta_{12}\mathrm{AB}+\beta_{13}\mathrm{AC}+\beta_{14}\mathrm{AD}+\beta_{23}\mathrm{BC}+\beta_{24}\mathrm{BD}+\beta_{34}\mathrm{CD}+\beta_{11}A^{2}\;+\beta_{22}B^{2}\;+\beta_{33}C^{2}\;+\beta_{44}D^{2}$$

where *Y* represents the dependent variable (SDF/IDF) and *β*_0_ is the intercept term. The independent variables are represented as *A* (cellulase addition), *B* (enzymatic hydrolysis time), *C* (alcoholic precipitation liquid ratio), and *D* (ultrasonic power). *β_i_* are the linear coefficients, *β_ij_* are the cross-product coefficients, and *β_ii_* are the quadratic coefficients.

The SDF and IDF obtained under the optimal conditions were used for subsequent experiments. 

### 2.3. Characterization of DF Structure

#### 2.3.1. Thermogravimetric Analysis (TGA) 

DF was pulverized and passed through a 200-mesh sieve. Samples of 10 mg were determined by a TGA analyzer (TGA55, TA Instruments Co., New Castle, DE, USA) with the protection of nitrogen at a rate of 40 mL/min. The samples were thermogravimetrically examined in the range of 20–600 °C with a warming rate of 10 °C/min. The results were expressed as TG and DTG curves.

#### 2.3.2. Fourier-Transform Infrared (FT-IR) Spectroscopy

DF was crushed and sieved. Potassium bromide (200 mg) was mixed and ground with dietary fiber (1 mg), then pressed into transparent flakes. An infrared spectrometer was used for the analysis (Spectrum 100, Perkin Elmer Co., Waltham, MA, USA) in the range of 400–4000 cm^−1^ with a resolution set at 2 cm^−1^.

#### 2.3.3. X-Ray Diffraction (XRD) Analysis

DF was pulverized and passed through a 200-mesh sieve. A sample of 20 mg was taken and spread evenly in the sample holder. The slide compacted the sample until the surface was tightly flat and tightly bound to the frame. The crystal types of the samples were analyzed using an X-ray instrument (XRD-7000, Shimadzu Co., Tokyo Metropolis, Japan). The scanning diffraction angle (2*θ*) was scanned from 10° to 70° in 0.02 increments at a constant speed of 2°/min, using a tube current of 30 mA and a pressure of 30 kV.

#### 2.3.4. Scanning Electron Microscopy (SEM)

DF was pulverized and passed through a 200-mesh sieve. A tiny quantity of the sample was dabbed onto the conductive adhesive. After gold plating by a vacuum sprayer, the microstructures of the two DFs magnified 500 and 5000 times were observed using SEM (3400-I, Hitachi Co., Tokyo Metropolis, Japan), with a low-vacuum accelerating potential of 10–15 kV.

### 2.4. Analysis of Glucose-Lowering Activity

#### 2.4.1. Glucose Adsorption Determination

Different dietary fiber samples of 0.25 g were weighed into 250 mL conical flasks and 50 mL of glucose solution with concentrations of 10, 50 and 100 mmol/L was added. The mixture was centrifuged (4000 rpm, 15 min) after shaking in a shaker at 37 °C for 6 h. The supernatant was then extracted from the sample [21]. The glucose mass concentration in the supernatant was determined by the DNS method (UV-5500PC, Shanghai Metash Instruments Co., Shanghai, China). The standard curve of glucose was y = 5.023x + 0.013, R^2^ = 0.9994. The adsorption amount of glucose was calculated as follows:

(4) $$Glucose\;adsorption\;capacity\left(mmol/g\right)=\frac{{(c}_{0}-c_{e})V}{m}\times 100$$

where *c*_0_ is the initial concentration of glucose solution (μmol/L), *c_e_* is the glucose concentration at adsorption equilibrium (μmol/L), *V* is the volume of glucose solution (L), and *m* is the quality of samples (g).

#### 2.4.2. α-Amylase Inhibition Assay

The following method was used for determination of *α*-amylase inhibitory activity [22]. Samples measuring 200 μL of different concentrations of sample solution, 200 μL of 0.5% (*w*/*v*) soluble starch solution, and 200 μL of 10 U/mL *α*-amylase solution were put into in test tubes and mixed well. After incubating at 37 °C for 10 min, 300 μL of supernatant was added with 400 μL of DNS reagent. After 5 min of heating in boiling water, the solution was chilled in ice water. Then 1 mL of PBS buffer (pH = 6.8) was added to it and the absorbance was measured at 540 nm (EPOCH12, Bio Tek Instruments Co., Winooski, VT, USA). For the sample control group, the enzyme solution was replaced with PBS. The blank control group used PBS instead of sample solution and enzyme solution. The positive control group used acarbose instead of sample solution. The inhibition rate was computed using the formula below:

(5) $$\alpha -amylase\;activity\;inhibition\;rate\left(\%\right)=\left(1-\frac{A_{1}-A_{2}}{A_{3}-A_{4}}\right)$$

where *A*_1_, *A*_2_, *A*_3_, and *A*_4_ are the absorbance for the sample group, sample control group, control group, and blank control group, respectively.

*α*-amylase inhibition kinetics. The concentration of soluble starch was fixed at 0.5%, the sample concentrations were selected (SDF: 0, 1, and 2 mg/mL; IDF: 0, 2, and 3 mg/mL), and the *α*-amylase concentrations were varied to 0.2, 0.4, 0.6, 0.8, and 1 U/mL. Based on the above conditions, the rate of enzyme reaction was determined. A relationship curve was plotted with enzyme concentration as the horizontal coordinate and reaction rate as the vertical to determine the reversibility of the inhibition of *α*-amylase by DF. The concentration of amylase was fixed at 1 U/mL, the sample concentrations were selected (SDF: 0, 1, and 2 mg/mL; IDF: 0, 2, and 3 mg/mL), and the concentration of starch was 0.1%, 0.2%, 0.3%, 0.4%, and 0.5%. Based on the above conditions, the rate of reaction was determined. The type of inhibition of *α*-amylase by DF was determined by plotting a Lineweaver–Burk double inverse plot.

#### 2.4.3. α-Glucosidase Inhibition Assay

The following method was used for determination of *α*-glucosidase inhibitory activity [23]. Samples measuring 200 μL of different concentrations of sample solution, 200 μL of 3 mmol/L PNPG, and 200 μL of 2800 U/mL of *α*-glucosidase solution were put into test tubes and mixed well. After incubating at 37 °C for 20 min, 750 μL of a 0.1 M Na_2_CO_3_ solution was added to terminate the reaction and absorbance was measured at 405 nm. Using acarbose as a positive control, the inhibition rate was computed using Formula (4).

*α*-Glucosidase inhibition kinetics. The concentration of the PNPG was fixed at 3 mmol/L, the sample concentrations were selected (SDF: 0, 1, and 3 mg/mL; IDF: 0, 4, and 8 mg/mL), and the concentrations of the α-glucosidase solution were 980, 1400, 2800, 4200, and 5600 U/mL. Based on the above conditions, the reaction rate was determined. The concentration of enzyme solution was plotted against the reaction rate, determining the reversibility of the inhibition of *α*-glucosidase by DF. The concentration of glycosidase was fixed at 2800 U/mL, the sample concentrations were selected (SDF: 0, 1, and 3 mg/mL, IDF: 0, 4, and 8 mg/mL), and the concentrations of the PNPG were 0.25, 0.50, 1.00, 1.50, and 2.00 mg/mL. Based on the above conditions, the reaction rate was determined. Lineweaver–Burk double inverse curves were plotted to determine the type of *α*-glucosidase inhibition by DF.

### 2.5. Statistical Analyses

Every experiment was conducted three times and the mean ± standard deviation was used to represent the findings. Data analysis was conducted using Design-Expert 8.0.6, Origin Pro 2021 software, and SPSS 25 software.

## 3. Results

### 3.1. Analysis of Ultrasound–Enzymatic Sequential Programme Design Tests

**Figure 2:**
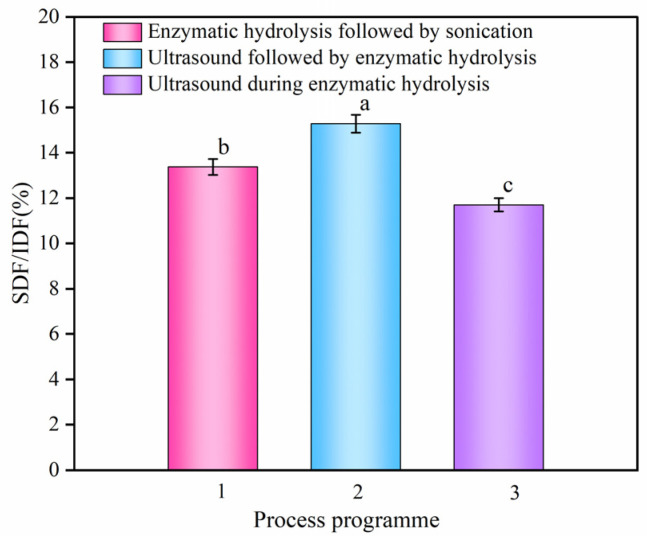
Effect of ultrasonic coupled enzyme process combination sequence on SDF/IDF ratio. SDF and IDF represent the soluble dietary fiber and insoluble dietary fiber, respectively. Different lowercase letters indicate that there are significant differences between the treatments according to the mean separation test (*p* < 0.05).

The physiological function of DF is related to the proportion of SDF and IDF. Most nutritionists recommended that the ratio of daily dietary fiber intake is 70–80% for IDF and 20–30% for SDF [24]. The common dietary fiber resources are dominated by IDF. The content of SDF is only 3% to 4% and it has low physiological activity. In contrast, high-quality dietary fiber composition should have more than 10% SDF [25], at which time it is more physiologically active and can be used as a high-quality fiber fortifier. As can be seen from Figure 2, three different process sequences significantly affected the SDF/IDF ratio extracted from sea buckthorn pomace. The comparison found that the combination with the highest SDF/IDF ratio was ultrasound followed by enzymatic hydrolysis. At this time, the process extraction efficiency was more advantageous. This is because ultrasound loosened the dense intercellular tissue structure and disrupted the plant cell wall [26]. Therefore, the contact area between the enzyme and the cell was increased during the enzymatic hydrolysis process, which accelerated the conversion of IDF to SDF by cellulase [27]. Ultimately, the SDF/IDF ratio was increased.

### 3.2. Analysis of Response Surface Optimization Experiment Results

The outcomes of single-factor experiments determined the primary factor parameters for the extraction of DF from sea buckthorn pomace as follows: cellulase addition of 5%, enzymatic hydrolysis time of 30 min, ultrasonic power of 380 W, and alcohol precipitation liquid ratio of 4:1. Taking the above results as the central point, the process parameters for the aforementioned factors were optimized using the response surface approach. The results of the experimental design are shown in Table 1. The analysis yielded the fitted quadratic multinomial regression equation:

(6) $$Y=17.07-0.0408A-0.0383B+0.4117C+0.3775D-0.0525AB+0.635AC+0.155AD+0.0525BC-0.02BD-0.4325CD-2.17A^{2}-0.8329B^{2}-1.7C^{2}-1.79D^{2}$$

The ANOVA is shown in Table 2. The overall model was highly significant (*p* < 0.01), R^2^ = 0.9796, and the lack of fit was not significant (*p* > 0.05). The results show that the model fits well and can be employed to ascertain the optimal extraction conditions. R_Adj_^2^ = 0.9782 indicated that the test was reliable and accurate. The factors of *C*, *D*, *A*^2^, *B*^2^, *C*^2^, *D*^2^, *AC*, and *CD* on the SDF/IDF yield were extremely significant (*p* < 0.01). From the *F*-value, the order of factors affecting the SDF/IDF ratio is *C* > *D* > *A* > *B*.

**Figure 3:**
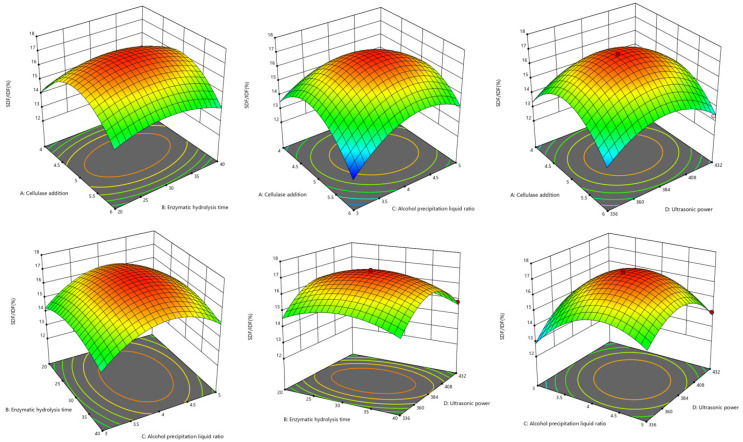
Response surface plots of factor interaction.

The results of the effect of each interaction term on the SDF/IDF ratio are shown in Figure 3. With the increase in enzyme addition, enzymatic hydrolysis time, and ultrasonic power, the SDF/IDF ratio increased first and then decreased. This result was consistent with that of the single-factor trials. The contour lines’ density and shape can reflect how the variables interact. The elliptical shape of the *AB* and *BD* contour lines indicate that the interactions between enzyme addition and enzymatic hydrolysis time, alcohol precipitation liquid ratio, and ultrasonic power were significant. The *BC* contour lines are relatively dense along the direction of the alcohol precipitation ratio, which indicates that the alcohol precipitation liquid ratio had a more significant effect on the SDF/IDF ratio.

The model was utilized to determine the optimal conditions for the ultrasonic–enzymatic extraction of DF from pomace: cellulase addition of 5.01%, ultrasonic power of 384.62 W, enzyme hydrolysis time of 29.79 min, and alcohol precipitation liquid ratio of 4.11:1. Under these conditions, the SDF/IDF ratio was 17.11%. Considering the actual situation, the process conditions were adjusted as follows: cellulase addition of 5%, ultrasonic power of 380 W, enzyme hydrolysis time of 30 min, and alcohol precipitation liquid ratio of 4:1. Under these conditions, the SDF yield of pomace was (13.34 ± 1.70)%, the IDF yield was (78.00 ± 2.10)%, and the SDF/IDF ratio was (17.07 ± 1.40)%. This result was 0.46% different from the model’s predicted value (17.11%). It indicates that the theoretical predicted value of the model fitted well with the actual value.

Under the same process conditions, the SDF yield was (9.25 ± 1.60)%, the IDF yield was (77.50 ± 1.80)%, and the SDF/IDF ratio was (11.91 ± 1.46)% when ultrasound was used alone. The SDF yield was (8.37 ± 1.40)%, the IDF yield was (77.92 ± 1.61)%, and the SDF/IDF ratio was (10.72 ± 1.29)% when cellulase was used alone. It can be seen that the quality and total yield of DF from sea buckthorn pomace prepared by the ultrasound–enzyme method were significantly superior.

### 3.3. Structural Characterization of Different DFs

#### 3.3.1. TGA Analysis 

**Figure 4:**
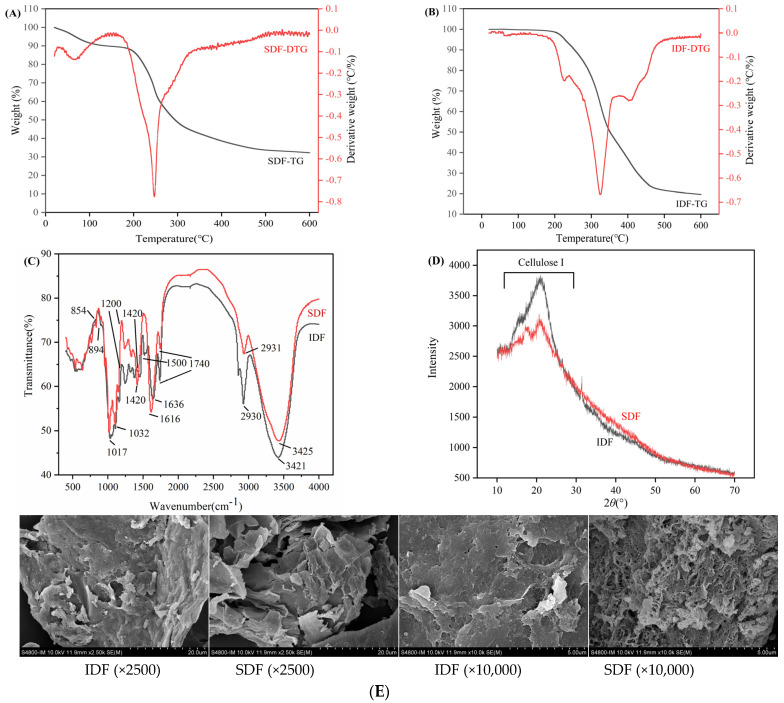
Structural characterization of different DFs. (**A**,**B**) TGA curves, (**C**) FT-IR, (**D**) X-ray diffraction and (**E**) SEM.

As shown in Figure 4A,B, the results of TG and DTG curves indicated the samples’ mass loss and mass loss rate versus temperature, respectively. The weightlessness processes of SDF and IDF all had three phases, the first of which was between 20 and 200 °C. DF lost weight slightly, and the weight loss rate of SDF was greater than that of IDF, which was mainly due to the volatilization of the intermolecular free water and bound water of DF [28]. Stage 2 was from 200 to 500 °C, during which cellulose and other substances were thermally decomposed, and the sample lost weight rapidly. The third stage was from 500 to 600 °C, during which the primary constituents of DF had undergone thermal degradation, while trace levels of lignin were gradually breaking down to yield ash and carbon [29]. The rate of pyrolysis tended to stabilize, and the residual mass of SDF was more significant than that of IDF. In summary, below 200 °C, IDF and SDF had good thermal stability. However, when the temperature exceeded 200 °C, the thermal stability of SDF and IDF showed significant heterogeneity, with SDF having better thermal stability. The greater the thermal stability, the more hydrophobic groups are exposed, allowing increased penetration of water in the dietary fiber molecules, and resulting in greater water-holding capacity and glucose adsorption capacity [30].

#### 3.3.2. FTIR Analysis

As illustrated in Figure 4C, the peaks of SDF and IDF were similar, indicating that the chemical compositions were similar. There were broad and smooth O-H stretching vibration peaks of SDF and IDF at 3425 cm^−1^ and 3421 cm^−1^, respectively. Furthermore, the absorption bands of IDF were wider, indicating that the intermolecular hydrogen bonding generated by the hydroxyl group of IDF was stronger than that of SDF. The C-H stretching vibration on the methyl and methylene groups of saccharides was responsible for the peaks at 2931 and 2929 cm^−1^. The peaks at 1636 and 1616 cm^−1^ corresponded to the O-H bending vibration, which was a characteristic peak for adsorbed water [31]. The C=O stretching vibration in the carboxylic acid group was shown by the absorption peak around 1740 cm^−1^, which behaved more strongly in IDF. This indicates that alduronic acid existed in an insoluble form in IDF. The aromatic ring’s distinctive stretching vibration peak in lignin is located close to 1500 cm^−1^, where the IDF peak was intense and there was almost no peak in SDF. This indicated that IDF was rich in lignin and SDF was almost free of lignin. Both curves show obvious absorption peaks in the range of 1420–1200 cm^−1^, which are characteristic absorption peaks of polysaccharides. This is produced by the variable angle vibration of C-H, and the peak of IDF is stronger. The SDF had an iconic absorption peak at 854 cm^−1^ for *α*-pyranose, while the IDF had an iconic absorption peak at 894 cm^−1^ for *β*-structure. In conclusion, both DFs possessed comparable functional groups and contained iconic absorption peaks for polysaccharides [32].

#### 3.3.3. XRD Analysis

As illustrated in Figure 4D, the X-ray diffraction curves of sea buckthorn pomace SDF and IDF are close. The 2*θ* positions at which the main peaks of X-ray diffraction appear were all between 20–30°. There were weak peaks near 17° and 31°, indicating that the crystal structure of both fibers was I.-type cellulose, and the two phases of crystalline and non-crystalline regions coexist. The peak shape of the IDF was more robust and sharper than that of SDF, which indicated that the crystal structure of IDF was orderly, the crystallinity was higher, and the water solvent could not easily penetrate. The molecular mass of SDF was small, and the degree of polymerization was low, which led to the weak strength of the cellulose and hemicellulose crystalline regions [31].

#### 3.3.4. SEM Analysis

As illustrated in Figure 4E, there were significant differences between the surfaces of samples. The microstructure of SDF is much looser and has a distinct honeycomb structure. SDF’s greater specific surface area exposes more hydrophilic–lipophilic groups, which could support its hydration and adsorption capabilities. IDF was partially glycosidic bond disrupted after ultrasound–enzymatic hydrolysis, and the surface was an irregular lamellar structure with numerous folds. IDF’s dense structure indicated that the adsorption performance of IDF might not be as good as that of SDF [33].

### 3.4. Glucose Adsorption by Different DFs

**Figure 5:**
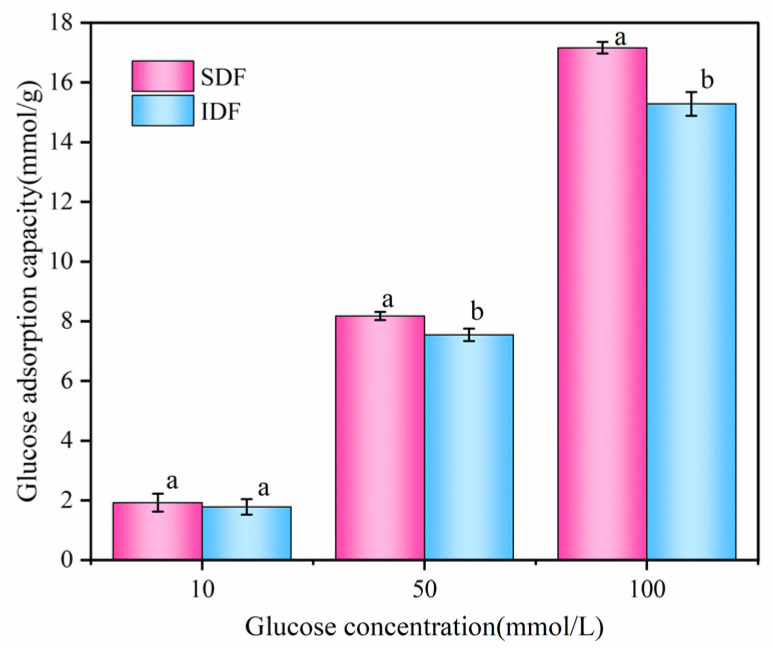
Glucose adsorption capacity of different DFs from sea buckthorn pomace. Different letters indicate that there are significant differences (*p* < 0.05).

As illustrated in Figure 5, the evaluation of the samples’ glucose adsorption capacity was conducted using three distinct concentrations of glucose solutions (10, 50, and 100 mmol/L). The adsorption capacity of SDF and IDF for glucose increased with increasing glucose concentrations. The functional groups presented in SDF and IDF, such as uronic acid and aldehydes, had strong affinity to glucose [34], and SDF adsorbed glucose better than IDF when the glucose concentration was the same. This is because the difference in glucose adsorption capacity was closely related to the surface morphology of DFs. When compared to IDF, the surface of SDF exhibited a honeycomb structure and lower molecular crystallinity, as demonstrated by the study of SEM and XRD. The porous surface and low crystallinity provide more binding sites for glucose, making it easier for glucose to enter the interior of SDF [35], which facilitated the adsorption and retention of glucose [36,37]. Moreover, the viscosity of SDF increased after water absorption, which substantially delays the body’s rate of absorbing glucose [38,39]. Zhang [30] found that SDF adsorbed glucose more strongly than IDF, which is consistent with the results of the current study.

### 3.5. Inhibition of Hypoglycemic Enzymes by Different DFs

#### 3.5.1. Inhibitory Activity on Hypoglycemic Enzymes

**Figure 6:**
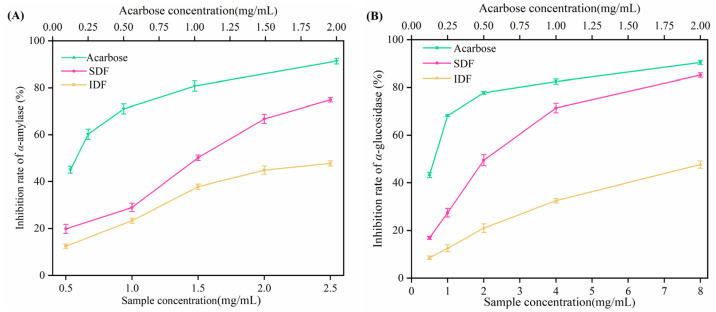
Effect of DFs from sea buckthorn pomace on glucose-lowering enzyme activity. (**A**) Inhibitory effects of DFs and acarbose on *α*-amylase, (**B**) Inhibitory effects of DFs and acarbose on *α*-glucosidase.

*α*-Amylase and *α*-glucosidase were the critical enzymes for carbohydrate hydrolysis. Decreasing their activities could help to delay the degree of blood glucose elevation [18]. As shown in Figure 6 and Table 3, the inhibitory effects of SDF and IDF on two enzymes both increased with increasing concentrations in a positive dose-dependent trend. The samples significantly inhibited hypoglycemic enzyme activities in the 0.5–2.5 mg/mL concentration range, using acarbose as a positive control. The IC_50_ data indicated that SDF inhibited both *α*-amylase and *α*-glucosidase more effectively than IDF. Additionally, the two pomace fibers inhibited *α*-amylase more than *α*-glucosidase. The structure of DF can affect how it inhibits enzymes. Firstly, the abundant hydroxyl groups in DF interact with the enzyme’s amino acid residue through hydrogen bonding, changing the enzyme’s structure by forming an enzyme–inhibitor–substrate complex [40]. Secondly, DF inhibits enzyme activity by encapsulating the enzyme and substrate in a porous or fibrous network structure [41]. SDF’s complex network and loose structure are more favorable to its encapsulation of substrates and enzymes. Therefore, SDF is more effective at inhibiting enzymes [42].

#### 3.5.2. Analysis of the Type of Inhibition of Hypoglycemic Enzymes

**Figure 7:**
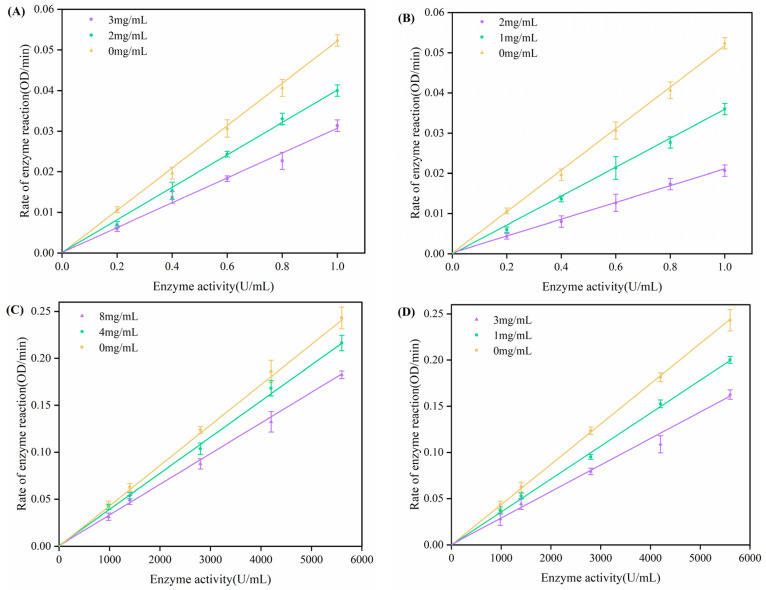
Inhibition reversibility of glucose-lowering enzyme by DFs from sea buckthorn pomace. (**A**) IDF—*α*-amylase, (**B**) SDF—*α*-amylase, (**C**) IDF—*α*-glucosidase, (**D**) SDF—*α*-glucosidase.

This experiment explored the inhibition types of *α*-glucosidase and *α*-amylase by sea buckthorn pomace SDF and IDF. As shown in Figure 7, the inhibition of the two hypoglycemic enzymes by different concentrations of SDF and IDF both had a linear relationship. The straight lines passed through the origin, showing that the inhibitor interacts with the enzyme via non-covalent bonding and reduces its activity. As the sample concentration rose, the line’s slope dropped. This indicated that the samples do not change the amount of enzyme, but the activity and catalytic efficiency of the enzymes decrease [43]. Therefore, the types of inhibitory effects of the two DFs on hypoglycemic enzymes were reversible. Enzyme activity can be restored by physically altering the structure of DF [44,45].

#### 3.5.3. Lineweaver–Burk Plots for Hypoglycemic Enzymes

**Figure 8:**
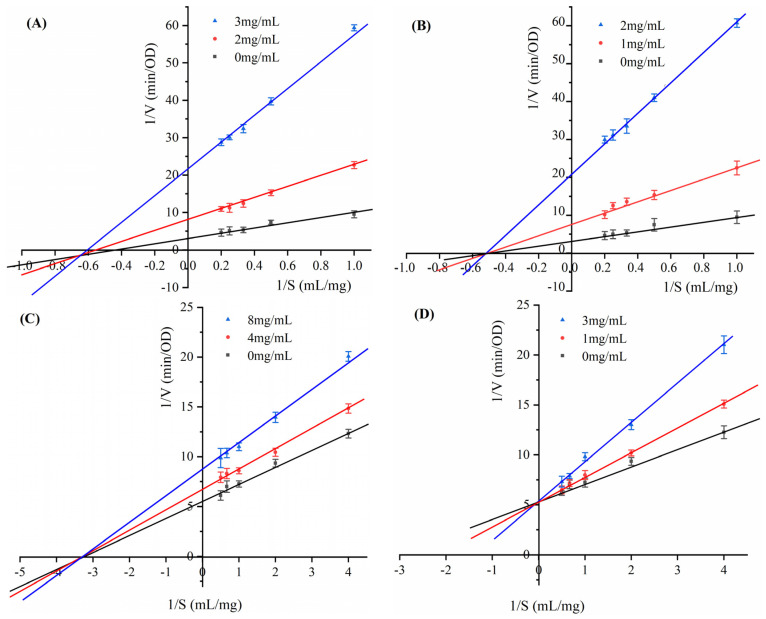
Lineweaver–Burk curve of sea buckthorn pomace DF on glucose-lowering enzyme. (**A**) IDF—*α*-amylase, (**B**) SDF—*α*-amylase, (**C**) IDF—*α*-glucosidase, (**D**) SDF—*α*-glucosidase.

The variation patterns of the Mie constant *K*_m_ and the maximum enzymatic rate *V*_max_ are analyzed using Lineweaver–Burk plots. The slope of the fitted line is *K*_m_/*V*_max_, the horizontal axis intercept is −1/*K*_m_, and the vertical axis intercept is 1/*V*_max_ [46]. As shown in Figure 8A, the fitted lines in the Lineweaver–Burk plot of the inhibition of *α*-amylase by IDF intersected at a point in the third quadrant. As the IDF concentration increased, both *K*_m_ and *V*_max_ showed a decreasing trend. Thus, the inhibition of *α*-amylase by IDF falls under the category of mixed inhibition [47]. As depicted in Figure 8B, the Lineweaver–Burk plot of the inhibition of *α*-amylase by SDF showed excellent linearity between 1/*V* and 1/*S*. The fitted lines intersected at a point on the negative half-axis of the *x*-axis. As the concentration of SDF increased, *K*_m_ remained unchanged and *V*_max_ decreased. This indicated that the inhibition type of SDF on *α*-amylase belongs to the non-competitive type of inhibition [48]. As can be seen from Figure 8C, the fitted lines of IDF with different concentrations intersected the negative half-axis of the *x*-axis. The longitudinal axis intercept (1/*V*_max_) increased with the increase in the concentration of IDF, while *K*_m_ remained unchanged and *V*_max_ decreased. The above characteristics were consistent with non-competitive inhibition. As illustrated in Figure 8D, the fitted lines of SDF intersected the *y*-axis. As the concentration of SDF increases, *K*_m_ becomes larger and *V*_max_ remains constant. Thus, SDF inhibited *α*-glucosidase through competitive inhibition [49].

## 4. Conclusions

This study focused on optimizing SDF/IDF using innovative ultrasonic and enzymatic technologies, resulting in an SDF yield of 13.34 ± 1.70%, an IDF yield of 78.00 ± 2.10%, and a high SDF/IDF ratio of 17.07 ± 1.4%. Compared to using a single extraction method alone, this method improved the quality of dietary fiber while ensuring the total dietary fiber yield. As a plant cellulose preparation process, the method has advantages over its technical promotion.

Current dietary fiber research focuses on the function of different IDF and SDF composite ratios. However, a certain range of ratios will produce distinct results with constraints. This study compares and analyzes the structural and hypoglycemic properties of SDF and IDF. It provides a reference for the independent study of IDF and SDF and theoretically proves that both have significant hypoglycemic effects. The IC_50_ of SDF for *α*-amylase was 56.63% of IDF’s, and that for *α*-glucosidase was 21.21% of IDF’s. At 100 mmol/L of glucose, the glucose adsorption capacity of SDF was 1.12 times greater than that of IDF. Thus, SDF and IDF are expected to have significant marketing potential, since they can be used as high-quality sources of cellulose in hypoglycemic foods and as raw ingredients in low-glycemic index foods (GI).

## Figures and Tables

**Table 1:** Box–Behnken design and results.

Run	*A* Cellulase Addition (%)	*B* Enzymatic Hydrolysis Time (min)	*C* Alcohol Precipitation Liquid Ratio (mL/mL)	*D* Ultrasonic Power (W)	SDF (%)	IDF (%)	SDF/IDF (%)
1	6	20	4:1	380	9.81 ± 0.52	69.18 ± 1.79	14.20 ± 1.01
2	4	30	3:1	380	9.56 ± 0.06	70.77 ± 4.33	13.54 ± 0.75
3	5	30	5:1	430	9.91 ± 0.10	70.45 ± 3.43	14.08 ± 0.59
4	4	40	4:1	380	9.70 ± 0.51	68.51 ± 2.81	14.19 ± 1.32
5	5	20	5:1	380	10.04 ± 1.36	68.22 ± 2.24	14.69 ± 1.56
6	5	30	3:1	330	7.39 ± 0.18	59.88 ± 3.84	12.36 ± 0.62
7	4	30	5:1	380	8.71 ± 0.41	66.73 ± 2.96	13.07 ± 0.71
8	5	40	4:1	430	10.73 ± 0.41	72.33 ± 2.74	14.85 ± 0.85
9	4	30	4:1	330	7.54 ± 0.47	59.47 ± 2.13	12.70 ± 1.12
10	5	40	5:1	380	10.65 ± 0.37	72.04 ± 3.24	14.81 ± 1.09
11	5	20	4:1	330	10.36 ± 0.48	73.06 ± 4.27	14.23 ± 1.36
12	5	20	3:1	380	9.96 ± 0.07	71.31 ± 3.89	14.00 ± 0.78
13	5	30	4:1	380	13.19 ± 0.65	77.61 ± 2.89	17.00 ± 0.55
14	4	30	4:1	430	8.63 ± 0.54	65.59 ± 3.57	13.19 ± 1.22
15	6	30	4:1	430	9.46 ± 0.15	70.48 ± 4.04	13.45 ± 0.62
16	5	30	4:1	380	13.11 ± 0.78	76.90 ± 2.27	17.05 ± 0.79
17	5	20	4:1	430	11.25 ± 0.39	75.62 ± 2.18	14.89 ± 0.81
18	4	20	4:1	380	10.12 ± 0.97	70.60 ± 1.09	14.33 ± 1.21
19	5	30	4:1	380	13.71± 0.47	79.50 ± 0.84	17.25 ± 0.57
20	5	30	5:1	330	9.74 ± 1.43	69.12 ± 3.12	14.10 ± 2.10
21	6	30	4:1	330	7.33 ± 0.35	59.54 ± 2.75	12.34 ± 1.15
22	6	40	4:1	380	9.49 ± 0.40	68.57 ± 2.03	13.85 ± 0.92
23	5	40	4:1	330	10.27 ± 0.92	71.86 ± 2.24	14.27 ± 0.84
24	6	30	5:1	380	10.56 ± 0.73	73.66 ± 3.67	14.38 ± 1.46
25	5	30	4:1	380	13.22 ± 0.81	77.88 ± 2.73	17.01 ± 1.51
26	5	30	3:1	430	10.08 ± 0.95	72.02 ± 3.70	14.07 ± 2.03
27	5	30	4:1	380	13.18 ± 0.31	77.81 ± 1.42	16.95 ± 0.66
28	6	30	3:1	380	7.59 ± 0.53	61.28 ± 3.18	12.31 ± 1.47
29	5	40	3:1	380	9.72 ± 0.57	69.91 ± 1.31	13.91 ± 0.84

**Table 2:** Analysis of variance of response surface method.

Variance Source	Sum of Squares	Degrees of Freedom	Mean Square	*F* Value	*p* Value	Significance
Model	57.46	14	4.10	95.29	<0.0001	**
*A* (Enzyme additions)	0.020	1	0.020	0.4645	0.5066	
*B* (Enzymatic hydrolysis time)	0.0176	1	0.0176	0.4094	0.5326	
*C* (Alcohol precipitation liquid ratio)	2.03	1	2.03	47.21	<0.0001	**
*D* (Ultrasonic power)	1.71	1	1.71	39.70	<0.0001	**
*AB*	0.011	1	0.011	0.256	0.6208	
*AC*	1.61	1	1.61	37.45	<0.0001	**
*AD*	0.0961	1	0.0961	2.23	0.1574	
*BC*	0.011	1	0.011	0.256	0.6208	
*BD*	0.0016	1	0.0016	0.0371	0.8499	
*CD*	0.7482	1	0.7482	17.37	0.0009	**
*A* ^2^	30.45	1	30.45	707.0	<0.0001	**
*B* ^2^	4.50	1	4.50	104.5	<0.0001	**
*C* ^2^	18.65	1	18.65	432.9	<0.0001	**
*D* ^2^	20.88	1	20.88	484.8	<0.0001	**
Residual	0.603	14	0.0431			
Lack of fit	0.5518	10	0.0552	4.31	0.0859	not significance
Pure error	0.0512	4	0.0128			
Sum	58.06	28				
	R^2^ = 0.9796	R_Adj_^2^ = 0.9782	A_deq_ precision = 33.1608		C.V% = 1.44	

Note: ** *p* < 0.01.

**Table 3:** IC_50_ values of hypoglycemic enzyme activities of DFs from sea buckthorn pomace.

Hypoglycemic Enzyme	Sample	IC_50_ (mg/mL)
*α*-Amylase	Acarbose	0.16 ^c^
	IDF	2.49 ^a^
	SDF	1.41 ^b^
*α*-Glucosidase	Acarbose	0.14 ^c^
	IDF	9.24 ^a^
	SDF	1.96 ^b^

Note: For data in the same column with the same enzyme, different letters indicate significant differences (*p* < 0.05).

## Data Availability

The original contributions presented in the study are included in the article, further inquiries can be directed to the corresponding authors.
